# Comparison of Tigecycline or Cefoperazone/Sulbactam therapy for bloodstream infection due to Carbapenem-resistant *Acinetobacter baumannii*

**DOI:** 10.1186/s13756-019-0502-x

**Published:** 2019-03-06

**Authors:** Tianshui Niu, Qixia Luo, Yaqing Li, Yanzi Zhou, Wei Yu, Yonghong Xiao

**Affiliations:** 1 Collaborative Initiative Center for Diagnosis and Treatment of Infectious Diseases, State Key Laboratory for Diagnosis and Treatment of Infectious Diseases, the First Affiliated Hospital, college of Medicine, Zhejiang University, Hangzhou, 310003 China; 2 Women’s Hospital School of Medicine Zhejiang University, Hangzhou, 310003 China; 3 Zhejiang Provincial People’s Hospital, People’s Hospital of Hangzhou Medical College, Hangzhou, 310003 China

**Keywords:** *Acinetobacter baumannii*, Carbapenem resistance, Tigecycline, Cefoperazone/sulbactam

## Abstract

**Background:**

We retrospectively analyzed the effect of tigecycline and cefoperazone/sulbactam therapies on the prognosis of patients with carbapenem-resistant *Acinetobacter baumannii* bloodstream infection (CRAB-BSI).

**Methods:**

CRAB-BSI patients receiving tigecycline therapy or cefoperazone/sulbactam therapy between January 2012 and December 2017 was enrolled, and strict exclusion criteria were followed. The 28-day mortality of patients was analyzed. The impact of cefoperazone/sulbactam therapy on prognosis was evaluated using Cox multivariate regression analysis. The 28-day mortality of patients receiving cefoperazone/sulbactam monotherapy and cefoperazone/sulbactam-based combination therapy was also compared.

**Results:**

Three hundred forty eight patients with CRAB-BSI were enrolled in the study. Two hundred ten patients were included after applying the exclusion criteria. Of these, 135 patients received tigecycline therapy and 75 patients received cefoperazone/sulbactam therapy. The 28-day mortality of patients in the latter group was, significantly lower than that of the tigecycline group [29.3% vs. 51.9%; *P* = 0.001]. Cox multivariate regression analysis revealed that cefoperazone/sulbactam therapy exerted a protective effect on the prognosis of patients [hazard ratio 0.566, 95% confidence interval (0.342–0.940); *P* = 0.028]. Kaplan-Meier survival curve analysis indicated that the 28-day mortality of patients receiving cefoperazone/sulbactam therapy was lower than that of patients receiving cefoperazone/sulbactam monotherapy, but the difference was not significant (22.2% vs. 40%; *P* = 0.074). However, the mortality of patients receiving cefoperazone/sulbactam with imipenem/cilastatin was significantly lower than that of patients receiving cefoperazone/sulbactam monotherapy (*P* = 0.048).

**Conclusions:**

Patients treated with cefoperazone/sulbactam therapy had a better clinical outcome. The mortality of patients receiving cefoperazone/sulbactam with imipenem/cilastatin seems to be the lowest.

**Electronic supplementary material:**

The online version of this article (10.1186/s13756-019-0502-x) contains supplementary material, which is available to authorized users.

## Background

*Acinetobacter baumannii* (AB) is one of the most important pathogens associated with hospital-acquired infections worldwide. It causes a wide range of infections, such as respiratory tract infection, blood infection, abdominal infections, urinary tract infections, traumatic infection, central nervous system infection, skin infections, which seriously threaten the health of patients [1, 2]. Because AB is highly resistant to many antibiotics and disinfectants, it is difficult to eliminate, and as such, it often becomes established in the hospital environment [3]. Carbapenem antibiotics are the first-line drugs for treating AB infections [4]. However, because of their widespread use, AB resistance to carbapenem antibiotics has rapidly increased, especially among strains isolated from the intensive care unit [5]. In the United States, the incidence of carbapenem-resistant AB (CRAB) increased from 20.6% in 2002 to 49.2% in 2008 [6]. In China, it increased from 31% in 2005 to 66.7% in 2014 [7]. Currently, very few drugs are available for the treatment of carbapenem-resistant AB (CRAB). In vitro, CRAB is highly sensitive to only a few drugs, such as polymyxin and tigecycline.

The best treatment for CRAB infection is currently unclear. In China, sulbactam-based combination therapy, tigecycline-based combination therapy, and polymyxin-based combination therapy are recommended for the treatment of multidrug resistant (MDR) Gram-negative bacilli [8]. However, these recommendations are based on small-scale retrospective studies, lacking systematic and comprehensive clinical research evidence, and no large-scale clinical randomized controlled trials have been performed to evaluate their efficacy in patients with MDR-AB. Because of the toxic side effects of polymyxin, the drug is not widely used in Mainland China [9]. Therefore, sulbactam therapy and tigecycline therapy are currently the main clinical treatments for CRAB. However, many controversies surround tigecycline regimen for treating AB bloodstream infections (BSI). The US Food and Drug Administration recommendated that tigecycline had been independently associated with a higher risk of mortality and should only be used in settings where therapeutic options were limited [10]. Tigecycline exerts a good therapeutic effect according to some studies, while numerous other studies reported that tigecycline increases patient’s mortality [11–13]. Therefore, it is important to identify the best treatment for CRAB-BSI.

In the current study, we analyzed clinical data from patients with CRAB-BSI, and compared the prognosis of patients receiving cefoperazone/sulbactam therapy and tigecycline therapy. We also analyzed the effect of cefoperazone/sulbactam monotherapy and combination therapy on the prognosis of patients to determine the optimal regimen for improving the clinical treatment effect.

## Methods

### Research design and patient selection

This study was conducted at the First Affliated Hospital, College of Medicine, Zhejiang University, after receiving approval from the research ethics committee (Reference Number: 2017–699). We were granted ethical approval for a waiver of informed consent and accessed the medical records of the patients considered for inclusion. Patients with CRAB were enrolled in the study from January 2012 to December 2017. Carbapenem resistance was defined as a minimum inhibitory concentration (MIC) of ≥8 μg/ml for imipenem and meropenem, according to the breakpoints of the Clinical and Laboratory Standards Institute (CLSI) standards [14]. Cefoperazone-sulbactam susceptibility was based on the breakpoints for ampicillin-sulbactam (MIC of 16/8 μg/ml) [15]. Tigecycline susceptibility was determined using the US Food and Drug Administration breakpoints [16]. Susceptibility to other drugs was determined according to the CLSI standards [14]. BSI was assessed by following the criteria proposed by the US Centers for Disease Control and Prevention. Patients were included if they had at least one AB-positive blood culture and symptomatic disease (fever [> 38 °C or < 36 °C], chills, hypotension, or other symptoms); if patients had more than one episode of AB-BSI, only data from the first episode were included. Tigecycline therapy was defined as tigecycline monotherapy or tigecycline with other antibiotics (including cefoperazone/sulbactam), with tigecycline doses of at least 50 mg every 12 h (q12h) for more than 48 h [17]. Cefoperazone/sulbactam therapy was defined as cefoperazone/sulbactam monotherapy or cefoperazone/sulbactam with other antibiotics (without tigecycline), of which the dose of cefoperazone/sulbactam (cefoperazone: sulbactam, 2:1) was 1 g q6h or q8h, or 2 g q6h or q8h for more than 48 h.

The exclusion criteria were as follows: patients with CRAB who died within 48 h or patients administered antibiotics for less than 48 h; patients for whom clinically critical data were missing; and patients receiving treatment regimens that included neither tigecycline therapy nor cefoperazone/sulbactam therapy. Patients were included in the study in one of these arms regardless of the results of cefoperazone/sulbactam or tigecycline susceptibility testing. The prognosis of patients with CRAB was based on 28-day mortality.

### Research

**Fig. 1:**
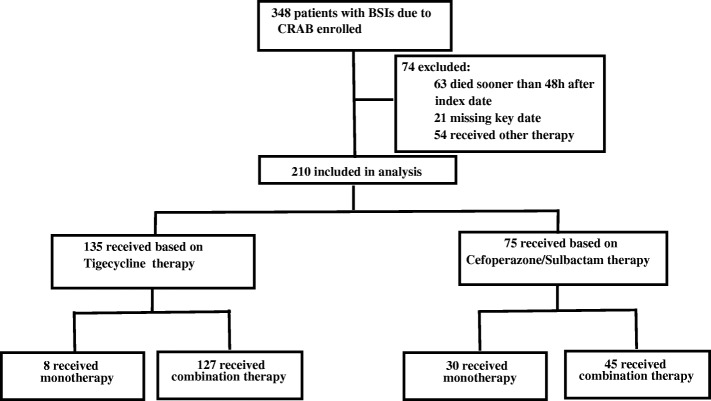
Case selection process

Two-step analysis was performed in the current study (Fig. 1). The effects of receiving tigecycline therapy and cefoperazone/sulbactam therapy on patients’ prognosis were first compared, and the patients were classed into low-risk (APACHE II < 20) and high-risk groups (APACHE II ≥ 20). The 28-day mortality of patients receiving tigecycline therapy and cefoperazone/sulbactam therapy in different risk groups was analyzed. Cox multivariate regression analysis was used to determine the impact of cefoperazone/sulbactam therapy on patient’s prognosis. Then, the 28-day mortality of patients receiving cefoperazone/sulbactam monotherapy and cefoperazone/sulbactam-based combination therapy was analyzed, and that of patients receiving cefoperazone/sulbactam monotherapy and cefoperazone/sulbactam-based combination therapy in different risk groups.

The following information was collected from the hospital information management system: demographic parameters, underlying disease, complications, vital signs, laboratory data on infection, acute physiology and chronic health assessment (APACHE II score), Pitt bacteremia score (PBS), clinical pulmonary infection score (CPIS), bacteriological tests, and use of antibiotics upon diagnosis of BSI.

### Statistical analysis

Statistical analysis was performed using SPSS22.0. Categorical variables were analyzed using the Chi-square or Fisher’s exact test., and continuous variables were analyzed using t-test and Wilcoxon rank-sum test. Cox regression analysis for multivariate analysis was used after evaluating the proportional hazard assumptions. Variables demonstrating a difference with a *P*-value of < 0.1 were included in the Cox regression analysis. Results from the Cox regression analysis were analysed and interpreted using a P-value of < 0.05 to indicate a statistically significant difference. Kaplan-Meier analysis was used to evaluate the survival curves of patients receiving different treatments. For the analyses, *P* < 0.05 was considered to indicate statistically significant difference.

## Results

### Demographic parameters and drug susceptibility testing

348 patients with CRAB infection were enrolled in the current study. After applying the exclusion criteria, 210 (60.3%) patients were included in the study. Of these, 135 patients (64.3%) received tigecycline therapy and 75 patients (35.7%) received cefoperazone/sulbactam-based therapy. The characteristics of patients receiving tigecycline therapy and cefoperazone/sulbactam therapy are compared in Table 1. The median age of patients receiving tigecycline therapy was 62 years (21–95 years), while the median age of patients receiving cefoperazone/sulbactam therapy was 60 years (3–85 years). In the two groups, approximately 70% of patients were male, and more than 70% of patients had been admitted to the intensive care unit during hospitalization. The median APACHE II score was higher in the tigecycline therapy group than in the cefoperazone/sulbactam therapy group [20 (9–33) vs. 18 (7–31)], but the difference was not significant. The median CPIS score was higher in the tigecycline therapy group than in the cefoperazone/sulbactam therapy group [7 (2–12) vs. 6 (2–10)]. Among 210 patients, 119 patients were secondary to lower respiratory tract infection, 35 patients were catheter-related infection and 26 patients were abdominal infection (Additional file 1:Table S1). The common underlying diseases in the two groups were hypertension, hepatitis B/cirrhosis. The common complications during hospitalization were pulmonary infection, septic shock, and respiratory failure.

**Table 1:** Characteristicsof CRAB-BSI patients with Tigecycline therapy and Cefoperazone/Sulbactam therapy

	Tigecycline therapy (*n* = 135)	Cefoperazone/Sulbactam therapy (*n* = 75)	*P* value
Age (years)	62 (21–95)	60 (3–85)	0.086
Male sex	94 (69.6%)	55 (73.3%)	0.571
ICU admission	101 (74.8%)	55 (73.3%)	0.814
APACHE II score	20 (9–33)	18 (7–31)	0.063
APACHE II score < 20	66 (48.9%)	48 (64%)	
APACHE II score ≥ 20	69 (51.1%)	27 (36%)	0.035
PBS	3 (0–7)	3 (0–8)	0.098
CPIS	7 (2–12)	6 (2–10)	0.006
28 day mortality	70 (51.9%)	22 (29.3%)	0.001
APACHE II score < 20	30.3% (20/66)	18.8%(9/48)	0.118
APACHE II score ≥ 20	72.5% (50/69)	48.1% (13/27)	0.023
Underlying disease			
hypertension	58 (43.0%)	23 (30.7%)	0.053
hepatitis/cirrhosis	24 (17.8%)	19 (25.3%)	0.131
diabetes	22 (16.3%)	12 (16.0%)	0.560
renal insufficiency	23 (17.0%)	11 (14.7%)	0.406
coronary	17 (12.6%)	9 (12%)	0.544
respiratory	17 (12.6%)	8 (10.7%)	0.431
tumor	9 (6.7%)	5 (6.7%)	0.622
Comorbid conditions			
pulmonary infection	51 (37.8%)	24 (32.0%)	0.247
septic shock	41 (30.4%)	5 (6.7%)	0.000
respiratory failure	24 (17.8%)	15 (20.0%)	0.412
MOF	16 (11.9%)	2 (2.7%)	0.017
abdominal cavity infection	12 (8.9%)	2 (2.7%)	0.069
stroke	3 (2.2%)	4 (5.3%)	0.208
gastrointestinal bleeding	5 (6.7%)	2 (2.7%)	0.515

Notes: Data are expressed as number (%) unless otherwise stated

Abbreviations: *CRAB-BSI* Acinetobacter baumannii bloodstream infection, *PBS* Pitt Bacteraemia Score, *CPIS* Clinical Pulmonary Infection Score APACHE II score, acute physiology and chronic health evaluation II, *ICU* intensive care unit; MOF, multiple organ failure

Drug sensitivity testing revealed that over 90% of AB isolated from patients were resistant to cefepime, ceftazidime, imipenem, meropenem, and ampicillin/sulbactam; 88.8% of AB isolates from the tigecycline therapy group and 72.7% of AB isolates from the cefoperazone/sulbactam therapy group were resistant to cefoperazone/sulbactam. The resistance of AB isolates to tigecycline was not as pronounced, with 14.7% of isolates from the tigecycline therapy group and 14.3% of isolates from the cefoperazone/sulbactam therapy group resistant to that antibiotic (Additional file 1:Table S2).

### Comparison of the 28-day mortality among different therapy groups

**Fig. 2:**
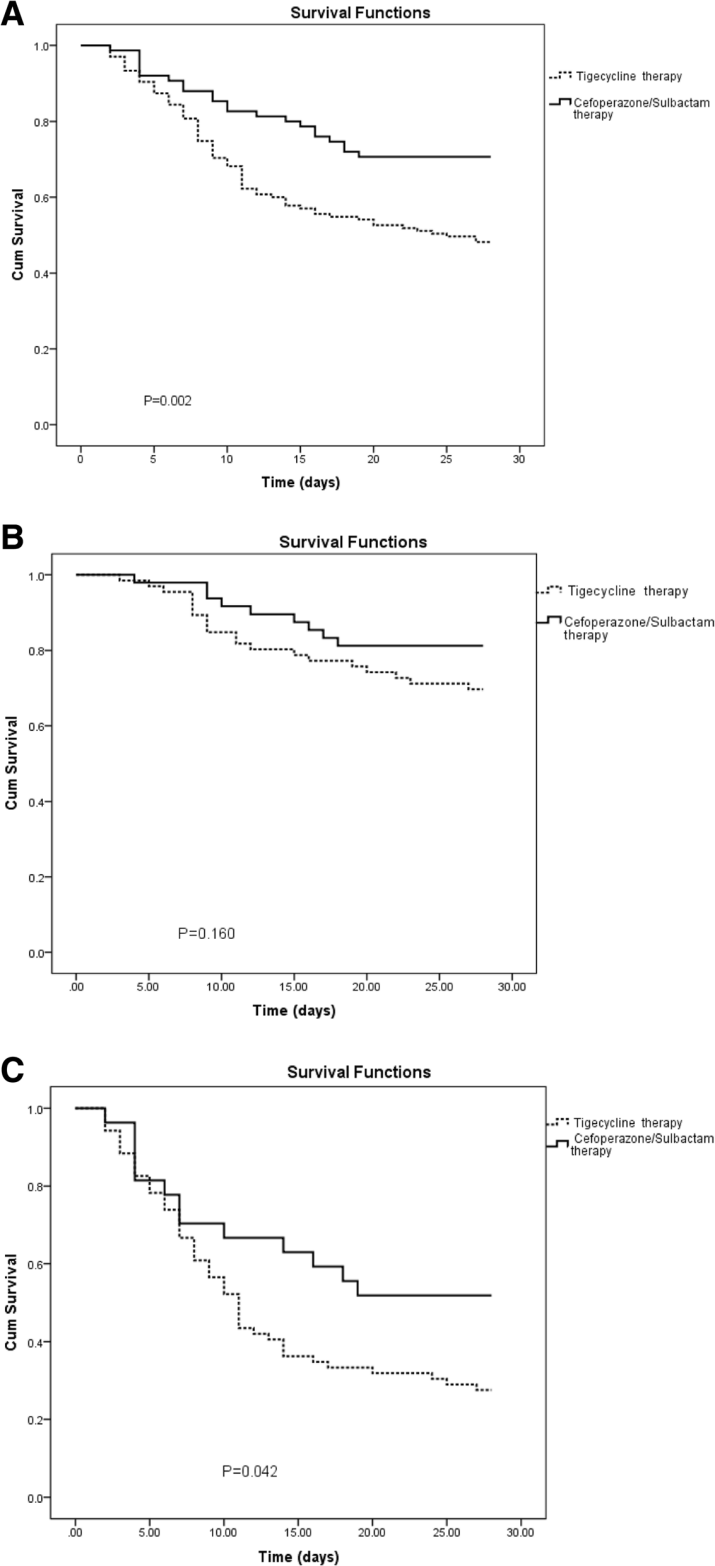
Kaplan-Meier survival estimates among CRAB-BSI patients. **a** Kaplan-Meier survival estimates among CRAB-BSI patients with Tigecycline therapy and Cefoperazone/Sulbactam therapy. **b** Kaplan-Meier survival estimates among CRAB-BSI patients (APACHE II score < 20) with Tigecycline therapy and Cefoperazone/Sulbactam therapy. **c** Kaplan-Meier survival estimates among CRAB-BSI patients (APACHE II score ≥ 20) with Tigecycline therapy and Cefoperazone/Sulbactam therapy. Abbreviations: CRAB-BSI, Acinetobacter baumannii bloodstream infection; APACHE II score, acute physiology and chronic health evaluation II

One hundred and thirty-five patients received tigecycline therapy, of which 70 patients (51.9%) died within 28 days (Table 1); and 75 patients received sulbactam therapy, of which 31 patients (41.3%) died within 28 days (Table 1). Further, the Kaplan-Meier survival curve revealed a significant reduction in the 28-day mortality (*P* = 0.002) in patients receiving cefoperazone/sulbactam compared with those receiving tigecycline (Fig. 2a). Patients receiving tigecycline were much more likely to have had septic shock (*P* = 0.000) and multi-organ failure (*P* = 0.017) (Table 1), this was consistent with the conclusion that patients receiving tigecycline have a higher mortality.

The patients were classed into low-risk and high-risk groups according to the APACHE II score (< 20 vs. ≥20). In the low-risk group, 66 patients (48.9%) received tigecycline therapy, with the 28-day mortality of 30.3% (20/66); while 48 patients (64.0%) received cefoperazone/sulbactam therapy, with the 28-day mortality of 18.8% (9/48) (Table 1). In the high-risk group, 69 patients (51.1%) received tigecycline therapy, with the 28-day mortality of 72.5% (50/69); and 27 patients (36.0%) received cefoperazone/sulbactam therapy, with the 28-day mortality of 48.1% (13/27) (Table 1). The Kaplan-Meier survival curve analysis revealed that in the high-risk group, the 28-day mortality of patients receiving cefoperazone/sulbactam therapy was significantly lower (*P* = 0.042) than that of patients receiving tigecycline therapy (Fig. 2b, Fig. 2c).

### Cefoperazone/sulbactam therapy is a protective factor for patient prognosis

A multivariate Cox logistic regression model was constructed. Univariate analysis indicated the following the *P* < 0.1 variables: APACHE II score ≥ 20, CPIS > 7, cefoperazone/sulbactam therapy, hypertension, multiple organ failure (MOF), and stroke. Multivariate Cox regression analysis revealed that the APACHE II score of ≥20 during hospitalization [hazard ratio (HR) = 2.530, 95% confidence interval (CI) (1.571–4.075); *P* = 0.000], CPIS > 7 [HR = 2.277, 95% CI (1.424–3.640); *P* = 0.001], and MOF [HR = 2.268, 95% CI (1.283–4.007); *P* = 0.005] were significantly associated with the 28-day mortality of patients. The cefoperazone/sulbactam therapy [HR = 0.566, 95% CI (0.342–0.940); *P* = 0.028] exerted a protective effect on the prognosis of patients (Table 2).

**Table 2:** Univariate and multivariate Cox regression analyses for mortality of patients with CRAB-BSI

	Crude analysis		Adjusted analysis	
	HR (95% CI)	*p* value	HR (95% CI)	*p* value
> 60 years of age	1.382 (0.782–2.443)	0.265	..	..
Male sex	0.817 (0.512–1.303)	0.395	..	..
ICU admission	1.610 (0.852–3.041)	0.143	..	..
APACHE II score ≧20 at infection	2.346 (1.197–4.600)	0.013	2.530 (1.571–4.075)	0.000
PBS > 3 at infection	0.773 (0.418–1.429)	0.411	..	..
CPIS > 7 at infection	2.107 (1.253–3.545)	0.005	2.277 (1.424–3.640)	0.001
Sulbactam therapy	0.592 (0.344–1.020)	0.059	0.566 (0.342–0.940)	0.028
Underlying disease				
hypertension	1.562 (0.324–2.973)	0.040	0.762 (0.496–1.171)	0.214
hepatitis/cirrhosis	1.004 (0.539–1.870)	0.991	..	..
diabetes	1.188 (0.640–2.204)	0.586	..	..
cardiac	1.188 (0.621–2.275)	0.602	..	..
respiratory	0.922 (0.481–1.764)	0.805	..	..
tumor	0.638 (0.224–1.817)	0.638	..	..
Comorbid conditions				
pulmonary infection	0.825 (0.514–1.324)	0.425	..	..
septic shock	1.431 (0.859–2.384)	0.169	..	..
respiratory failure	1.035 (0.600–1.784)	0.902	..	..
MOF	2.671 (1.399–5.103)	0.003	2.268 (1.283–4.007)	0.005
abdominal cavity infection	0.788 (0.331–1.877)	0.590	..	..
stroke	2.412 (0.875–6.648)	0.089	1.789 (0.697–4.588)	0.226
gastrointestinal bleeding	1.401 (0.537–3.653)	0.491	..	..

Note: “..”P < 0.1 in the univariate analysis were entered into a multivariate analysis; P ≥ 0.1 in the univariate analysis were not entered into a multivariate analysis

Abbreviations: CRAB-BSI, Acinetobacter baumannii bloodstream infection; HR, hazard ratio; CI, confidence interval;

*PBS* Pitt Bacteraemia Score, *CPIS* Clinical Pulmonary Infection Score APACHE II score, acute physiology and chronic health evaluation II, *ICU* intensive care unit, MOF, multiple organ failure

### The effect of cefoperazone/sulbactam monotherapy and combination therapy on patient prognosis

**Fig. 3:**
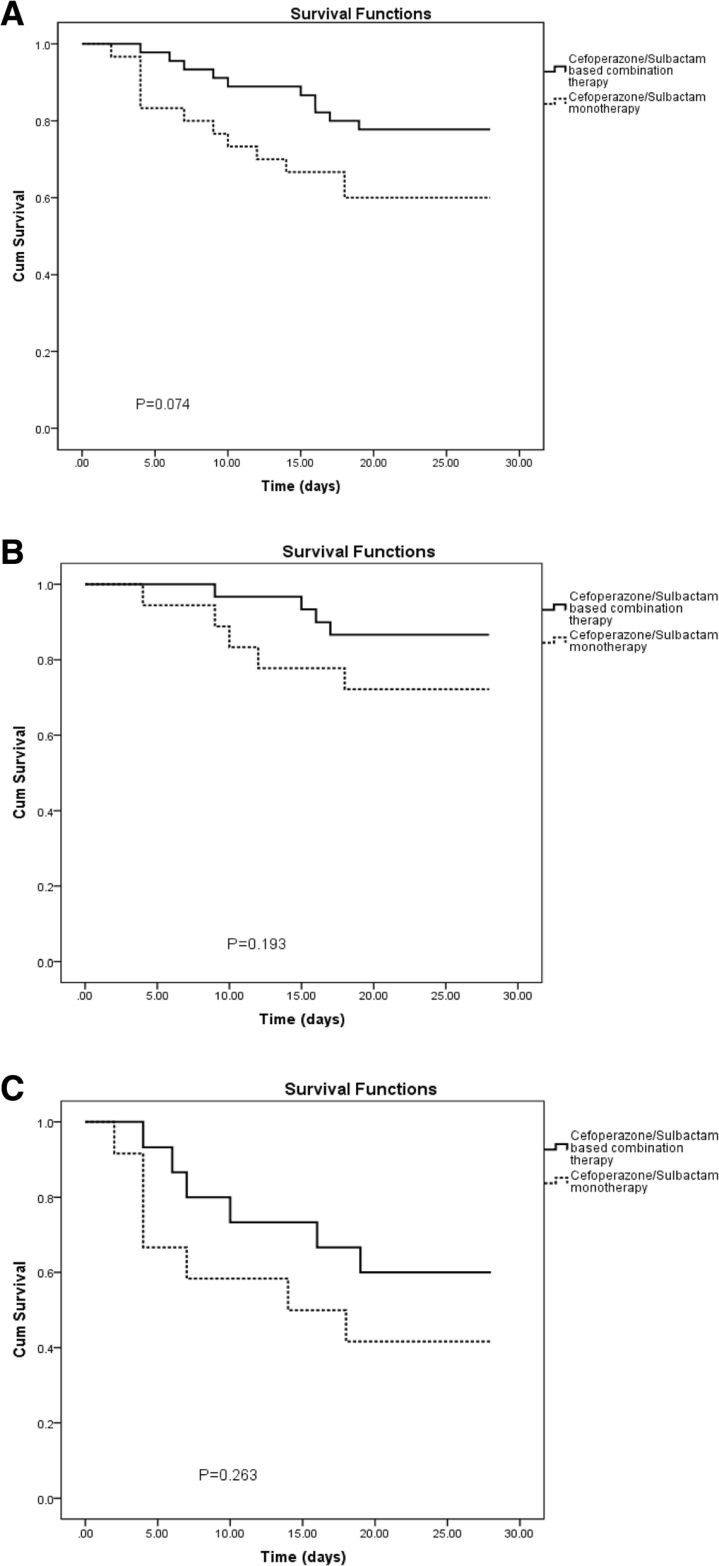
Kaplan-Meier survival estimates among CRAB-BSI patients with Cefoperazone/Sulbactam therapy. **a** Kaplan-Meier survival estimates among CRAB-BSI patients with Cefoperazone/Sulbactam monotherapy and Cefoperazone/Sulbactam based combination therapy. **b** Kaplan-Meier survival estimates among CRAB-BSI patients (APACHE II score < 20) with Cefoperazone/Sulbactam monotherapy and Cefoperazone/Sulbactam based combination therapy. **c** Kaplan-Meier survival estimates among CRAB-BSI patients (APACHE II score ≥ 20) with Cefoperazone/Sulbactam monotherapy and Cefoperazone/Sulbactam based combination therapy. Abbreviations: CRAB-BSI, Acinetobacter baumannii bloodstream infection; APACHE II score, acute physiology and chronic health evaluation II

Seventy-five patients received cefoperazone/sulbactam therapy, of which 30 patients (40%) received monotherapy and 45 patients (60%) received combination therapy (Table 3). The median APACHE II score of the cefoperazone/sulbactam monotherapy group was higher than that of the cefoperazone/sulbactam combination therapy group [19 (11–31) vs. 18 (12–31), respectively], but the difference was not significant. The 28-day mortality of patients receiving cefoperazone/sulbactam monotherapy was 40% (12/30), while that of patients received combination therapy was 22.2% (10/45) (*P* = 0.082) (Table 3). Further, the Kaplan-Meier survival curve revealed that the 28-day mortality in the cefoperazone/sulbactam combination therapy group was lower than that in the cefoperazone/sulbactam monotherapy group, but the difference was not statistically significant (*P* = 0.074) (Fig. 3a).

**Table 3:** Characteristics of CRAB-BSI patients with Cefoperazone/Sulbactam monotherapy and Cefoperazone/Sulbactam based combination therapy

	Cefoperazone/Sulbactam monotherapy (*n* = 30)	Cefoperazone/Sulbactam based combination therapy (*n* = 45)	*P* value
Age (years)	62 (21–95)	60 (3–85)	0.086
Male sex	20 (66.7%)	35 (77.8%)	0.211
ICU admission	21 (70.0%)	34 (75.6%)	0.392
APACHE II score	19 (11–31)	18 (12–31)	0.371
APACHE II score < 20	18 (60.0%)	30 (66.7%)	
APACHE II score ≥ 20	12 (40.0%)	15 (33.3%)	0.364
PBS	3 (2–8)	3 (1–6)	0.554
CPIS	6 (3–10)	6 (4–10)	0.969
28 day mortality	12 (40.0%)	10 (22.2%)	0.082
APACHE II score < 20	27.8% (5/18)	13.3% (4/30)	0.194
APACHE II score ≥ 20	58.3% (7/12)	40% (6/15)	0.288
Underlying disease			
hypertension	13 (43.3%)	10 (22.2%)	0.046
hepatitis/cirrhosis	8 (26.7%)	11 (24.4%)	0.518
diabetes	6 (20.0%)	6 (13.3%)	0.323
renal insufficiency	4 (13.3%)	7 (15.6%)	0.533
coronary	2 (6.7%)	7 (15.6%)	0.216
respiratory	0 (0%)	8 (17.8%)	0.013
tumor	2 (6.7%)	3 (6.7%)	0.687
Comorbid conditions			
pulmonary infection	7 (23.3%)	17 (37.8%)	0.144
septic shock	2 (6.7%)	3 (6.7%)	0.687
respiratory failure	8 (26.7%)	7 (15.5%)	0.188
MOF	1 (3.3%)	1 (2.2%)	0.643
abdominal cavity infection	1 (3.3%)	1 (2.2%)	0.643
stroke	2 (6.7%)	2 (4.4%)	0.527
gastrointestinal bleeding	1 (3.3%)	1 (2.2%)	0.643

Notes: Data are expressed as number (%) unless otherwise stated

Abbreviations: *CRAB-BSI* Acinetobacter baumannii bloodstream infection, *PBS* Pitt Bacteraemia Score, *CPIS* Clinical Pulmonary Infection Score APACHE II score, acute physiology and chronic health evaluation II, *ICU* intensive care unit, *MOF* multiple organ failure

In the low-risk group, 18 patients received cefoperazone/sulbactam monotherapy, with the 28-day mortality of 27.8% (5/18); and 30 patients received cefoperazone/sulbactam combination therapy, with the 28-day mortality of 13.3% (4/30) (Table 3). In the high-risk group, 12 patients received cefoperazone/sulbactam monotherapy, with the 28-day mortality of 58.3% (7/12); and 15 patients received cefoperazone/sulbactam combination therapy, with the 28-day mortality of 40.0% (6/15) (Table 3). The Kaplan-Meier survival curve analysis revealed that the 28-day mortality of patients receiving cefoperazone/sulbactam therapy was lower than that of patients receiving cefoperazone/sulbactam monotherapy, but the difference was not significant (Fig. 3b, Fig. 3c).

In the cefoperazone/sulbactam combination therapy group, the common combination regimen was cefoperazone/sulbactam with imipenem/cilastatin (55.6%, 25/45), and cefoperazone/sulbactam with biapenem or meropenem (22.2%, 10/45). The 28-day mortality of patients receiving cefoperazone/sulbactam with imipenem/cilastatin was lower than that of patients receiving the cefoperazone/sulbactam monotherapy (16% vs. 40%, respectively) (*P* = 0.048) (Table 4).

**Table 4:** 28 day mortality of Cefoperazone/Sulbactam monotherapy group and Cefoperazone/Sulbactam based combination therapy group

Treatment (Number)	Treatment (Number)	28 day mortality	p value
Sulbactam monotherapy (30)	Cefoperazone/Sulbactam (30)	40% (12/30)	
Sulbactam based combination therapy (45)	Cefoperazone/Sulbactam+ Imipenem/cilastatin (25)	16% (4/25)	0.048^*^
	Cefoperazone/Sulbactam+ Biapenem or Meropenem (10)	33.3% (3/10)	0.432
	Cefoperazone/Sulbactam+ Other antibiotic(10)	33.3% (3/10)	0.432

Note: “^*^” Asterisks indicate statistically significantly different from Cefoperazone/Sulbactam alone treatment (Chi-square test)

## Discussion

The mechanism of AB resistance is complex, which led to the increasing prevalence of MDR-AB [18, 19]. Drug-resistant AB infections are closely associated with increased patient mortality, the length of hospital stay, and hospitalization costs [20–22]. Currently, most AB isolates are resistant to first-line antibiotics and the effectiveness of tigecycline is controversial; the efficacy has been proven. Therefore, it is particularly urgent to explore the potential of the existing antibiotics and that of a combination therapy involving the existing antibiotics for AB treatment.

Sulbactam is a synthetic, irreversibly competitive sulbactam that has shown good clinical efficacy since its introduction [23]. Sulbactam is often combined with a β-lactam antibiotic, such as cefoperazone or ampicillin, to enhance its bactericidal action against MDR-AB. The cefoperazone/sulbactam combination is effective against AB infections [24]. Choi et al. reported that the 30-day mortality of patients with CRAB receiving cefoperazone/sulbactam [7/35 (20%)] was lower than that of patients with CRAB receiving imipenem/cilastatin [vs. 6/12 (50%), *P* = 0.065] [25]. Xia et al. reported that the 30-day survival rate of patients treated with cefoperazone/sulbactam or a cefoperazone/sulbactam combination regimen was significantly higher than that of patients who had not received cefoperazone/sulbactam (96.4% vs. 73.3%, respectively; *P* < 0.05), among patients with hospital-acquired pneumonia caused by CRAB [26].

However, AB resistance to sulbactam continues to increase with the extensive use of sulbactam. A survey in the United States demonstrated that the incidence of AB strains resistant to ampicillin/sulbactam rose from 35.2% in 2003–2005 to 41.2% in 2009–2012 [27]. AB resistance to cefoperazone/sulbactam in China increased from 25% in 2005 to 37.7% in 2014 [7]. In 2005, the US Food and Drug Administration approved tigecycline for treatment of complex abdominal infections, and complex skin and soft tissue infections, including complex appendicitis, burn infections, abdominal abscesses, deep soft tissue infections, and ulcer infections [28]. Because of its pronounced antibacterial activity and because a variety of bacteria are highly susceptible toward tigecycline, tigecycline is considered to be an off-label treatment for infections caused by MDR pathogens when the drug selection is limited.

Tigecycline is a commonly used drug for the treatment of pneumonia caused by AB resistant to carbapenem and other antibiotics, with a clinical curative effect of 60–88% [29–31]. However, tigecycline increases patient’s mortality. A meta-analysis of 14 randomized trials involving 7400 patients indicated no benefit of using tigecycline for treating severe infections compared with the use of standard antibiotics. In that study, the success rate of tigecycline treatment was lower than that of the control group [32]. Prasad et al. showed that tigecycline increases mortality (*P* = 0.01) and noncure rate (*P* = 0.01) [33]. The efficacy of sulbactam had also been compared with that of tigecycline. Ye et al. investigated pneumonia caused by MDR-AB, and reported no significant difference in the 30-day mortality between sulbactam group (17.9%) and tigecycline group (25.0%) patients (*P* = 0.259) [34]. Liang et al. reported that although tigecycline is often used to treat CRAB-induced pneumonia, tigecycline-based regimen is associated with increased mortality and failure rates. The mortality for a tigecycline-based regimen was 40.9% (65/159), while that of a sulbactam-based regimen was 8.3% (1/12) [35]. However, these studies focused on patients with pneumonia caused by CRAB, CRAB specimens mostly originated from the respiratory tract, and the sample size was small. Little is known about the clinical effects of different treatment regimens on CRAB-BSI.

In the current study, we compared the clinical effects of a tigecycline regimen with those of a cefoperazone/sulbactam regimen in detail. We found that 64.3% of patients followed the tigecycline regimen and only 35.7% patients followed the cefoperazone/sulbactam regimen, but the 28-day mortality in the latter group (35.7%) was lower than that in the former group (51.9%; *P* = 0.001). We also found that in the high-risk risk group (APACHE II score ≥ 20), 69 patients (51.1%) received tigecycline therapy, while 27 patients (36.0%) received cefoperazone/sulbactam therapy. However, the 28-day mortality in the sulbactam-treated group was lower than that in the tigecycline therapy group (48.1% vs. 72.5%, respectively; *P* = 0.042). The Cox multivariate regression analysis indicated that the cefoperazone/sulbactam regimen exerted a protective effect on the patient’s prognosis [HR = 0.566, 95% CI (0.342–0.940); *P* = 0.028]. Therefore, we believe that although the AB resistance rate to sulbactam is increasing, the sulbactam regimen continues to have a good therapeutic effect on CRAB-BSI. Although tigecycline shows pronounced antibacterial activity in vitro and is widely distributed in human tissues, its concentration in the serum is very low. The first dose of tigecycline is 100 mg, and it is followed by 50 mg every 12 h. The peak plasma concentration of tigecycline (C_max_) was reported to be only 0.87 μg/ml, with the minimum concentration (C_min_) only 0.13 μg/ml [36]. This impacts the antibacterial effect of tigecycline in the body. The antibacterial effect of tigecycline in vivo is not effective, and patients are more likely to develop septic shock leading to multiple organ failure and death, so there may be a higher mortality. We also found CPIS score was noted to be statistically significantly different on multivariate analysis, previously study indicated that patients with respiratory sources of infection may do poorly on tigecycline therapy as these infections are usually associated with a high inoculum of bacteria, which may have been a factor in the higher mortality in receiving tigecycline [37] . At the same time, using univariate analysis, we previously showed that tigecycline use is associated with carbapenem resistance in AB [38].

In the current study, patients receiving tigecycline with cefoperazone/sulbactam were classed as a tigecycline-treated group. Tigecycline is rarely used alone, and is often combined with cefoperazone/sulbactam or other antibiotics. We observed that the 28-day mortality in patients receiving tigecycline with cefoperazone/sulbactam was higher than that in patients receiving cefoperazone/sulbactam (50% vs. 29.3%, respectively; *P* = 0.06) (Additional file 1:Tables S3 and S4). Additionally, We also found that patients receiving combination therapy with tigecycline and an antibiotic other than cefoperazone/sulbactam or a carbapenem, had the highest mortality overall among patients receiving tigecycline-based combination therapy (53.3%) although the sample size was limited (Additional file 1:Table S3).

Currently, a combination therapy is used for treating MDR-AB [39], and the efficacy of sulbactam monotherapy and combination therapy has been reported. Kuo et al. compared treatment regimens for CRAB-BSI and found that the 30-day mortality of patients receiving ampicillin/sulbactam combined with carbapenem antibiotics was lower [8/26 (31%)] than that of patients receiving ampicillin/sulbactam monotherapy [2/5 (40%)], carbapenem antibiotics monotherapy [7/12 (58%)], and carbapenem antibiotics combined with amikacin [5/10 (50%)] [40]. Data presented in the current study suggest that the mortality of patients receiving cefoperazone/sulbactam combination therapy is lower than that of patients receiving cefoperazone/sulbactam monotherapy in the low-risk group (13.3% vs. 27.8%, respectively) and in the high-risk group (40.0% vs. 58.3%, respectively), but the differences were not significant. The sample sizes within the low and high risk groups in the cefoperazone/sulbactam group were small which may have resulted in non-statistically significant differences.

The 28-day mortality of patients treated with cefoperazone/sulbactam with imipenem/cilastatin was significantly lower than that of patients receiving cefoperazone/sulbactam monotherapy (*P* = 0.048). We believe that although AB is highly resistant to sulbactam and carbapenem antibiotics, receiving cefoperazone/sulbactam with imipenem/cilastatin has a good therapeutic effect as a routine regimen. Therefore, we recommend a combination therapy of cefoperazone / sulbactam and imipenem / cilastatin, even if the patient was infected with carbapenem or cefoperazone/sulbactam resistant *Acinetobacter baumannii*. This was mainly because the combination of imipenem / cilastatin and cefoperazone / sulbactam had a good synergistic effect, pharmacokinetic activity, clearance rate for severe bacterial infection [41–43].

The current study has some limitations. First, respiratory tract or other organ infections are common in patients with BSI. The current study focused on CRAB-BSI without a comprehensive assessment of the clinical impact of infections caused by other pathogens or BSI. Second, some patients enrolled in the current study had been transferred from other hospitals. AB-BSI may have occurred if antibiotics were used before the transfer; however, we were unable to collect detailed information on antibiotic use before the transfer, which may have impacted the outcome. Thirdly, this study is a retrospective study, we recommend that future research in the form of a clinical trial may be indicated to more firmly establish the role of cefoperazone/sulbactam in the treatment of CRAB-BSI, as research up to this point has largely been based on retrospective observational data.

## Conclusions

In conclusion, a detailed comparison of the use of cefoperazone/sulbactam therapy and tigecycline therapy against CRAB revealed that mortality was lower in both high and low risk groups with the use of cefoperazone/sulbactam, but that the include population was small. Cox analysis indicated that the cefoperazone/sulbactam therapy exerts a protective effect on the patient’s prognosis. We also found that the mortality of patients receiving cefoperazone/sulbactam with imipenem/cilastatin was lower than that of patients receiving cefoperazone/sulbactam monotherapy, and the difference was significant. These observations are of great significance and serve as a reference for clinical treatment.

## Additional file

Additional file 1: **Table S1.** Source distribution of 210 strains of CRAB-BSI. **Table S2.** Drug resistance of *A. baumannii.* **Table S3.** 28 day mortality of Tigecycline monotherapy group and Tigecycline based combination therapy group. **Table S4.** 28 day mortality among CRAB-BSI patients with Tigecycline+Cefoperazone/Sulbactam and Sulbactam based combination therapy. (DOC 54 kb)

## Abbreviations

APACHE II score: Acute physiology and chronic health evaluation II
CI: Confidence interval
CPIS: Clinical Pulmonary Infection Score
CRAB-BSI: Acinetobacter baumannii bloodstream infection
HR: Hazard ratio
ICU: Intensive care unit
MOF: Multiple organ failure
PBS: Pitt Bacteraemia Score
