# Supplementary material for: Comparison of Tigecycline or Cefoperazone/Sulbactam therapy for bloodstream infection due to Carbapenem-resistant Acinetobacter baumannii
Source: Antimicrob Resist Infect Control. 2019 Mar 6;8:52. doi: 10.1186/s13756-019-0502-x (PMC6404342; doi:10.1186/s13756-019-0502-x)
Supplement: Supplementary file 1 — Table S1. Source distribution of 210 strains of CRAB-BSI. Table S2. Drug resistance of A. baumannii. Table S3. 28 day mortality of Tigecycline monotherapy group and Tigecycline based combination therapy group. Table S4. 28 day mortality among CRAB-BSI patients with Tigecycline+Cefoperazone/Sulbactam and Sulbactam based combination therapy. (DOC 54 kb) [file 13756_2019_502_MOESM1_ESM.doc]

**TableS1: Source distribution of 210 strains of CRAB-BSI**

| Source distribution | Strains Number | Composition ratio (%) |
| --- | --- | --- |
| Lower respiratory tract infection | 119 | 56.7 |
| Catheter infection | 35 | 16.7 |
| Abdominal infection | 26 | 12.4 |
| Traumatic infection | 17 | 8.1 |
| Urinary system infection | 3 | 1.4 |
| Other infection | 10 | 4.8 |

Abbreviations: CRAB-BSI, Acinetobacter baumannii bloodstream infection

**Table S2: Drug resistance of *A. baumannii***

| Drug | A. baumannii from patients received Tigecycline therapy | | | A. baumannii from patients received Sulbactam therapy | | |
| --- | --- | --- | --- | --- | --- | --- |
|  | Sensitive (%) | Intermediate (%) | Resistance (%) | Sensitive (%) | Intermediate (%) | Resistance (%) |
| Ampicillin-  Sulbactam | 0 | 7.4 | 92.6 | 5.3 | 2.7 | 92 |
| Cefoperazone-Sulbactam | 2 | 9.2 | 88.8 | 10.9 | 16.4 | 72.7 |
| Cefepime | 0 | 0 | 100 | 2.7 | 0 | 97.3 |
| Ceftazidime | 1.5 | 1.5 | 97 | 6.7 | 0 | 93.3 |
| Imipenem | 0 | 0 | 100 | 0 | 0 | 100 |
| Meropenem | 0 | 0 | 100 | 0 | 0 | 100 |
| Levofloxacin | 3 | 44.4 | 52.6 | 4 | 41.3 | 54.7 |
| Amikacin | 29.6 | 3.7 | 66.7 | 40 | 1.3 | 58.7 |
| Gentamicin | 17.8 | 0 | 82.2 | 24 | 2.7 | 73.3 |
| Tigecycline | 71.6 | 13.7 | 14.7 | 59.2 | 26.5 | 14.3 |

**Table S3: 28 day mortality of Tigecycline monotherapy group and Tigecycline based combination therapy group**

|  | Treatment | 28 day mortality |
| --- | --- | --- |
| Tigecycline monotherapy | Tigecycline | 75% (6/8) |
| Tigecycline based combination therapy | Tigecycline+Cefoperazone/Sulbactam | 50% (41/82) |
|  | Tigecycline+  carbapenem (3/10) | 50% (15/30) |
|  | Tigecycline+  Other antibiotic(3/10) | 53.3% (8/15) |

**Table S4: 28 day mortality among CRAB-BSI patients with Tigecycline+Cefoperazone/Sulbactam and Sulbactam based combination therapy**

|  | 28 day mortality |
| --- | --- |
| Tigecycline+Cefoperazone/Sulbactam | 50% (41/82) |
| Sulbactam based combination therapy | 29.3% (22/75) |
|  | P=0.006 |
